# *Cpt1a* gene expression in peripheral blood mononuclear cells as an early biomarker of diet-related metabolic alterations

**DOI:** 10.3402/fnr.v60.33554

**Published:** 2016-11-23

**Authors:** Rubén Díaz-Rúa, Andreu Palou, Paula Oliver

**Affiliations:** Laboratory of Molecular Biology, Nutrition and Biotechnology, Universitat de les Illes Balears and CIBER de Fisiopatología de la Obesidad y Nutrición (CIBERobn), Palma de Mallorca, Spain

**Keywords:** dietary markers, prevention markers, PBMC, high-fat diet, high-protein diet, unbalanced diets, metabolic syndrome, liver steatosis

## Abstract

**Background:**

Research on biomarkers that provide early information about the development of future metabolic alterations is an emerging discipline. Gene expression analysis in peripheral blood mononuclear cells (PBMC) is a promising tool to identify subjects at risk of developing diet-related diseases.

**Objective:**

We analysed PBMC expression of key energy homeostasis-related genes in a time-course analysis in order to find out early markers of metabolic alterations due to sustained intake of high-fat (HF) and high-protein (HP) diets.

**Design:**

We administered HF and HP diets (4 months) to adult Wistar rats in isocaloric conditions to a control diet, mainly to avoid overweight associated with the intake of hyperlipidic diets and, thus, to be able to characterise markers of metabolically obese normal-weight (MONW) syndrome. PBMC samples were collected at different time points of dietary treatment and expression of relevant energy homeostatic genes analysed by real-time reverse transcription-polymerase chain reaction. Serum parameters related with metabolic syndrome, as well as fat deposition in liver, were also analysed.

**Results:**

The most outstanding results were those obtained for the expression of the lipolytic gene carnitine palmitoyltransferase 1a (*Cpt1a*). *Cpt1a* expression in PBMC increased after only 1 month of exposure to both unbalanced diets, and this increased expression was maintained thereafter. Interestingly, in the case of the HF diet, *Cpt1a* expression was altered even in the absence of increased body weight but correlated with alterations such as higher insulin resistance, alteration of serum lipid profile and, particularly, increased fat deposition in liver, a feature characteristic of metabolic syndrome, which was even observed in animals fed with HP diet.

**Conclusions:**

We propose *Cpt1a* gene expression analysis in PBMC as an early biomarker of metabolic alterations associated with MONW phenotype due to the intake of isocaloric HF diets, as well as a marker of increased risk of metabolic diseases associated with the intake of HF or HP diets.

An unbalanced proportion of macronutrients in the diet (mainly fats and proteins) is associated with alterations in body composition, metabolic parameters and different pathologies (1–6). In different animal and human models, alterations in the macronutrient composition of a diet are described as greatly relevant in energy homeostasis and metabolic disorders (7, 8). Increased fat consumption is related to obesity and metabolic complications, even when the subjects do not develop overweight (9). There is a special, and very prevalent, group of individuals, the metabolically obese normal-weight (MONW), who are not obese regarding weight and height (body mass index – BMI), but present higher visceral adiposity with ectopic fat deposition in tissues, such as liver, and alteration in parameters related to metabolic syndrome, such as insulin resistance (9–11). On the other hand, increased protein consumption in detriment of carbohydrates, which has become a popular strategy for body weight control, has also been related to a potential increase in health risk, especially in the long term (5, 6, 12–14).

One of the main efforts in nutritional research is to develop an early intervention for the identification and prevention of diet-related diseases such as diabetes mellitus type 2 and cardiovascular disease (15). For this, the study of new biomarkers of intake of unbalanced diets is very important because they can be used as indicators of metabolic or physiological deviations that may increase the risk of future chronic diseases related to unhealthy dietary habits (16). A fraction of blood cells, the peripheral blood mononuclear cells (PBMC), mainly composed by lymphocytes and monocytes/macrophages, is increasingly being used in nutritional studies as a source of biomarkers (17). These cells are easily obtainable by a simple blood extraction (18) and, in addition to their role in the innate immune system response, they can be used to assess biological responses as their gene expression profile may reflect the pathological and physiological state of the organism in humans and rats (19–21). PBMC travel around the body interacting with serum metabolites, thus they can reflect the gene expression patterns of different organs without needing invasive techniques (22).

We have previously shown that PBMC can reflect nutritional responses of key energy homeostatic tissues as well as metabolic deviations related to obesity development (19, 20, 23, 24). The use of techniques based on transcriptomic analysis can help provide more information on eventual biomarkers of certain physiophatological changes associated with impaired nutrition (25, 26). Recently, using microarray analysis, we have also shown that PBMC can reflect increased health risk due to long-term intake of diets with unbalanced macronutrient composition (14). These results encouraged us to search for early biomarkers in PBMC to detect metabolic deviations and health consequences of diets with an unbalanced macronutrient composition. Our research focused on the identification of early biomarkers to detect increased metabolic risk related to the normal-weight obesity syndrome due to the intake of isocaloric high-fat (HF) or high-protein (HP) diets. To this purpose, we designed a pair-fed isocaloric experiment in rats fed during 4 months with unbalanced HF and HP diets. We collected blood for isolation of PBMC, monthly and at the end of the experiment, in order to evaluate early changes in mRNA expression of key energy metabolism genes which could predict future health risks, before changes in others classical markers could be detected. Fatty liver is a leading cause of later chronic liver disease and is related to increased risk of death (27); however, there is a lack of non-invasive biomarkers for liver steatosis. Thus, we were particularly interested in identifying easily obtainable early biomarkers which could predict increased liver fatty deposition.

## Methods and materials

### Ethical approval

All experimental procedures followed in this study were reviewed and approved by the Ethical Committee of the University of the Balearic Islands, and guidelines for the use and care of laboratory animals of the university were followed.

### Animals

The experiments were conducted with 2-month-old male Wistar rats (Charles River Laboratories España, SA, Barcelona, Spain), single-housed at 22°C with a period of light/dark of 12 h and pair-fed with different experimental diets for 4 months. We chose 2-month-old rats to begin the experiment because our objective was to analyse the effect of the intake of unbalanced diets during adulthood. Two experimental procedures were performed. In the first experiment (Experiment 1), animals were divided into three groups: a control group (*n*=7), an HF group (HF60, *n*=7) and an HP group (HP, *n*=6). Control animals were fed a normolipidic diet (D12450B, Research Diets) containing 70% of energy (Kcal) from carbohydrates, 10% from fats and 20% from proteins. HF60 animals were fed an HF diet (D12492, Research Diets) containing 20% of energy from carbohydrates, 60% from fats (40% saturated and 60% unsaturated fat) and 20% from proteins. HP animals were fed an HP diet (Research Diets) containing 45% of energy from carbohydrates, 10% from fats and 45% from proteins (mainly casein). Diets were purchased from Brogaarden (Gentofte, Denmark). The same set of animals was used in a previous study aimed to characterise the physiological implications of the intake of unbalanced diets on body weight, adiposity, serum parameters and gene expression in key energy homeostatic tissues (28) and to determine by microarray analysis their effect on PBMC gene expression (14). These previous studies only addressed changes found at the end of the study (6-month-old animals), whereas the focus of the present work is on changes seen during the dietary intervention. In addition, we performed a second experiment (Experiment 2) with a new group of animals fed an HF diet but with a lower percentage of fat (45% instead of 60% Kcal). In this second experimental approach, 2-month-old animals were divided into two groups: a control group (*n*=6) and a HF group (HF45, *n*=5). Control animals were fed with the same diet used in Experiment 1, and HF45 animals were fed an HF diet (D12451, Research Diets) containing 35% of energy from carbohydrates, 45% from fats and 20% from proteins.

During the experimental trials, food was administered isocalorically. The HF60 and the HP groups in Experiment 1 and the HF45 group in Experiment 2 received an equal amount of Kcal as consumed by their controls the day before. The energy density of the diets used for calculation was as follows: control: 3.85; HF60: 5.24; HF45: 4.73 and HP: 3.85 Kcal per gram. When present, residual food in each cage was weighed, discarded and replaced with fresh diet every 24 h. Food was administered always at the same hour (13:00 h) and was available to the animals over a 24-h period. Food intake of all groups was recorded daily to calculate the daily caloric intake and cumulative caloric intake throughout the experiment; body weight was recorded three times a week.

At the end of the experimental period, animals were sacrificed by decapitation. Anaesthesia was not used in order to avoid interferences, and trunk blood was collected from the neck, stored at room temperature for 1 h and centrifuged at 3,000 rpm for 10 min at 4°C to collect serum. Liver and different white adipose tissue depots, both visceral (epididymal, mesenteric and retroperitoneal) and subcutaneous (inguinal), were rapidly removed and weighed.

### Blood extraction and PBMC isolation

After initiation of the experimental trials, blood samples were collected every month during the 4 months of the dietary treatment; thus, a total of four time points were available from each dietary group, corresponding to 3, 4, 5 and 6-month-old animals. Blood samples were obtained from the saphena vein in the post-prandial absorptive state within the two first hours of the light cycle (8.00–10.00 h) to isolate serum and PBMC. We spent around 5–10 min per sample collection and obtained 1–1.5 mL of blood per animal. For PBMC isolation, blood was collected using heparin in NaCl (0.9%) as an anticoagulant, and then cells were obtained by Ficoll gradient separation, according to the instructions indicated by the manufacturer (GE Healthcare Bio Sciences, Barcelona, Spain), with some modifications, as previously described (14). One week prior to sacrifice and at 4 months of age, animals were submitted to a nocturnal 14-h fast to collect serum in fasted conditions to calculate the homeostatic model assessment for insulin resistance (HOMA-IR) index.

### Body composition

Body composition (fat and lean mass) was measured every 15 days using an EchoMRI-700™ (Echo Medical Systems, LLC, TX, USA). Animals were not anaesthetised.

### Quantification of circulating glucose, insulin, triacylglycerols and non-esterified free fatty acids

Blood glucose was measured using an Accu-Chek Glucometer (Roche Diagnostics, Barcelona, Spain). Serum insulin and tumor necrosis factor (TNF)-alpha were measured using enzyme-linked immunosorbent assay (ELISA) kits from DRG Instruments, Marburg, Germany (insulin) and R&D Systems Europe, Abingdon, UK (TNF-alpha). Commercial enzymatic colorimetric kits were used for the determination of triacylglycerols (TG) (Sigma Diagnostics, St Louis, MO, USA) and non-esterified free fatty acids (NEFA) (Wako Chemicals GmbH, Neuss, Germany).

### HOMA-IR analysis

Insulin resistance was assessed by the HOMA-IR in rats submitted to overnight (14 h) fasting using the following formula of Matthews et al. (29). HOMA-IR=fasting glucose (mmol/L)×fasting insulin (mU/L)/22.5.

### Liver lipid and triacylglycerol determination

Quantification of total lipid levels was determined by the method of Folch et al. (30). Liver TG content was measured in liver lipid extracts as previously described (31) using the Serum Triglyceride Determination Kit (Sigma Aldrich, Madrid, Spain).

### Real-time RT-PCR analysis

Gene expression of key genes involved in energy homeostatic control (mainly lipid metabolism but also carbohydrate metabolism) was determined in the PBMC and liver by real-time reverse transcription-polymerase chain reaction (RT-PCR). Total RNA from PBMC and liver samples was extracted using Tripure Reagent (Roche Diagnostics, Barcelona, Spain). PBMC RNA was purified with Qiagen RNesay Mini Kit spin columns (Izasa SA, Barcelona, Spain), and liver RNA using E.Z.N.A. Total RNA Kit I (Omega Bio-Tek, Norcross, GA, USA) and treated with DNase I (Omega Bio-Tek). RNA yield was quantified on a Nanodrop ND 1000 spectrophotometer (NanoDrop Technologies, Wilmington, DE, USA), and its integrity was measured on an Agilent 2100 Bioanalyzer with RNA 6000 Nano chips (Agilent Technologies, South Queensferry, UK). Fifty nanogram for PBMC and 250 ng for liver of total RNA (in a final volume of 5 µL) were denatured at 65°C for 10 min and then reverse transcribed to cDNA using murine leukaemia virus reverse transcriptase (Applied Biosystems, Madrid, Spain) at 20°C for 15 min, 42°C for 30 min, with a final step of 5 min at 95°C in an Applied Biosystems 2720 Thermal Cycler (Applied Biosystems, Madrid, Spain). PCRs were performed from diluted (1/5 for PBMC and 1/20 for liver) cDNA template, forward and reverse primers (in a final concentration of 0.2 µm each) and Power SYBER Green PCR Master Mix (Applied Biosystems, CA, USA) in a total volume of 11 µL, with the following profile: 10 min at 95°C, followed by a total of 40 temperature cycles (15 sec at 95°C and 1 min at 60°C). Primers for the different genes analysed are described in Supplementary Table 1. All primers were obtained from Sigma Genosys (Sigma Aldrich Química SA, Madrid, Spain). To verify the purity of the products, a melting curve was produced after each run according to the manufacturer's instructions. The threshold cycle (Ct) was calculated by the instrument's software (StepOne Software v2.0), and the relative expression of each mRNA was calculated as a percentage versus control rats, using the 2^−ΔΔCt^ method (32). Data were normalised against the reference genes *beta-actin* and *integrin beta 1*. *Beta-actin* was used in liver because it is a well-known reference gene, and *integrin beta 1* was used in PBMC because microarray analysis performed with these samples at the final point of Experiment 1 (data not shown) showed equal and high expression for this gene in the different experimental groups.

### Statistical analysis

All data are expressed as the mean±SEM. Differences between HF or HP groups with controls were analysed using Student's *t*-test. In addition, a one-way analysis of variance (ANOVA) and least significant difference *post hoc* tests were performed in Experiment 1 to compare the three different experimental groups. The test used for each comparison is indicated in the footnote of the specific figures. Linear relationships between key variables were tested using Pearson correlation coefficients. All analyses were performed with SPSS for Windows (SPSS, Chicago, IL, USA). Threshold of significance was defined at *p*<0.05 and is indicated when different.

## Results

### Body weight and adiposity

**Fig. 1:**
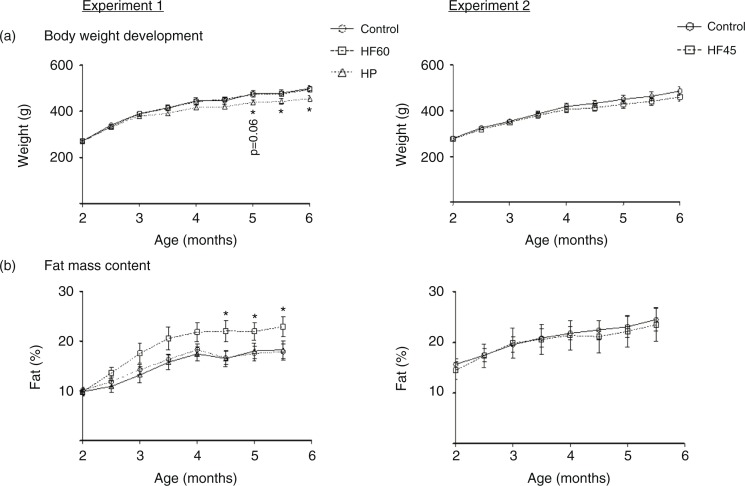
Body weight and fat mass content in animals of different ages fed a control, an HF60 or an HP diet (Experiment 1), or a control or an HF45 diet (Experiment 2). Diets were administered to 2-month-old Wistar rats during 4 months. HF and HP diets were offered in isocaloric amounts to control animals and water was offered *ad libitum*. Body weight was followed thrice a week. Results represent mean±SEM (*n*=5–7). *indicates values significantly different versus control animals (Student's *t*-test, *p*<0.05 or indicated when different).

As expected, at the end of the experimental trial, 6-month-old Wistar rats that had been isocalorically fed with the unbalanced HF diets (HF60 and HF45) for 4 months showed the same body weight as their respective controls (Fig. 1a). However, in spite of no difference in body weight, HF60 animals presented increased fat mass content (mainly due to the increase in visceral fat – data not shown), which was evident soon after the start of the dietary treatment and which became significant since day 134 of age (Fig. 1b). Nevertheless, no increase in fat mass was observed in the HF45 group (Fig. 1b). In the case of the HP group, rats presented a tendency to lower body weight throughout the 4 months of dietary treatment, which became significant since day 149 (Fig. 1a). This lower body weight was not related to any apparent decrease in fat mass (Fig. 1b) but mainly related to lower water content (228±6 g of total water in the HP vs. 253±8 g in the control group at the final time-point, *p*<0.05, Student's *t*-test). The cumulative energy intake was similar for the HF60 and HF45 animals and their controls, while HP animals showed a lower intake (data not shown).

### Indicators of glucose metabolism: circulating glucose, insulin and HOMA-IR index

**Fig. 2:**
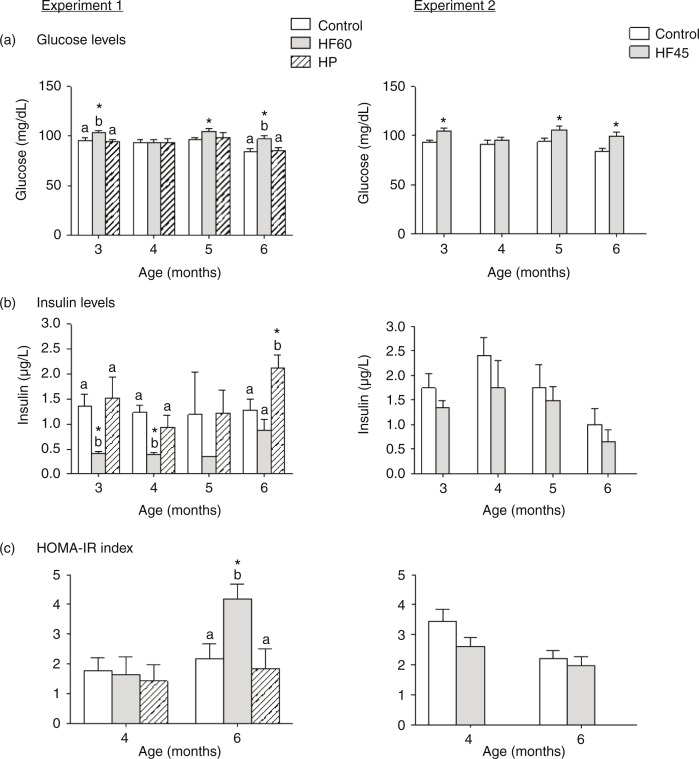
Circulating glucose, insulin levels and HOMA-IR index measured in the same animals and conditions described in Fig. 1. All parameters were measured in the fed state. Results represent mean±SEM (*n*=5–7). Values not sharing a common letter (a, b) are significantly different (one-way ANOVA, *p*<0.05); no letter indicates no significant differences; *indicates values significantly different versus control animals (Student's *t*-test, *p*<0.05).

In *ad libitum* conditions, the intake of both HF diets resulted in higher circulating glucose levels (Fig. 2a). This hyperglycaemia was observed from the beginning, after only 1 month of HF feeding (animals of 3 months of age), and throughout the dietary treatment. In contrast, serum insulin levels were lower for the first 2 months after intake of the HF60 diet, but were not affected in the HF45 group (Fig. 2b). Regarding the HP group, no changes were observed in circulating glucose or insulin levels, except at the end of the experiment, after 4 months of diet, when insulin levels were increased in the HP-fed animals (Fig. 2a and b). HOMA-IR index, measured at 2 and 4 months of dietary treatment (4- and 6-month-old rats, respectively), was increased only in animals of the HF60 group, and only at the end of the experiment, after 4 months of diet (Fig. 2c). No increase in HOMA-IR index was observed in the HF45 or in the HP groups.

### Indicators of lipid metabolism: circulating TG and NEFA, and TG and lipid content in liver

**Fig. 3:**
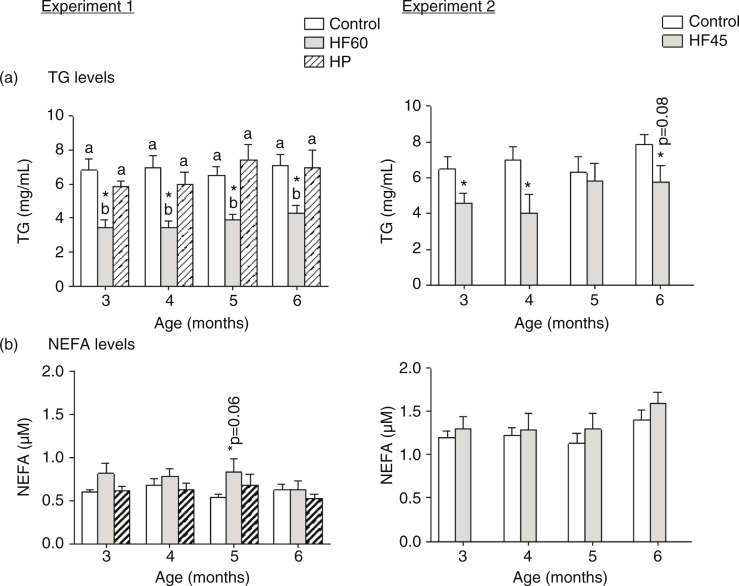
Circulating triacylglycerols (TG) and non-esterified fatty acids (NEFA) measured in the same animals and conditions described in Fig. 1. All parameters were measured in the fed state. Results represent mean±SEM (*n*=5–7). Values not sharing a common letter (a, b) are significantly different (one-way ANOVA, *p*<0.05); no letter indicates no significant differences; *indicates values significantly different versus control animals (Student's *t*-test, *p*<0.05 or indicated when different).

As shown in Fig. 3, both HF diets (HF60 and HF45) decreased serum TG levels in the fed state in the different months analysed. On the other hand, we observed a tendency to higher circulating NEFA levels in the two HF groups, but it did not attain statistical significance. Neither serum TG nor NEFA levels were affected by the HP diet.

**Fig. 4:**
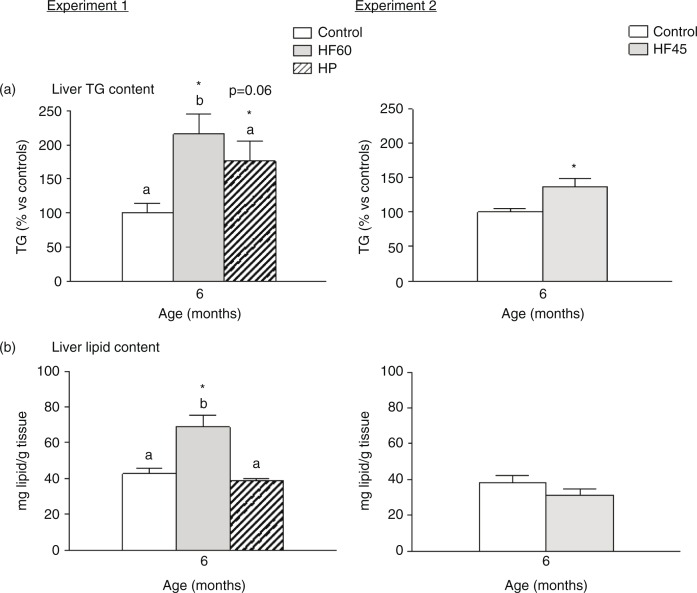
Liver triacylglycerol (TG) and total lipid content in 6-month-old animals fed during 4 months a control, an HF60 or an HP diet (Experiment 1), or a control or an HF45 diet (Experiment 2). All diets were offered in isocaloric amounts to control animals and water was offered *ad libitum*. Values not sharing a common letter (a, b) are significantly different (one-way ANOVA, *p*<0.05); no letter indicates no significant differences; *indicates values significantly different versus control animals (Student's *t*-test, *p*<0.05 or indicated when different).

Interestingly, the three unbalanced diets (HF60, HF45 and HP) increased TG content in liver, and the HF60 diet also increased total hepatic lipid content (Fig. 4). This is of relevance as fat deposition in liver is linked to systemic insulin resistance and the development of metabolic syndrome (33).

### Regulation of Cpt1a mRNA levels in PBMC and liver of rats fed with different unbalanced diets rich in fat or protein

**Fig. 5:**
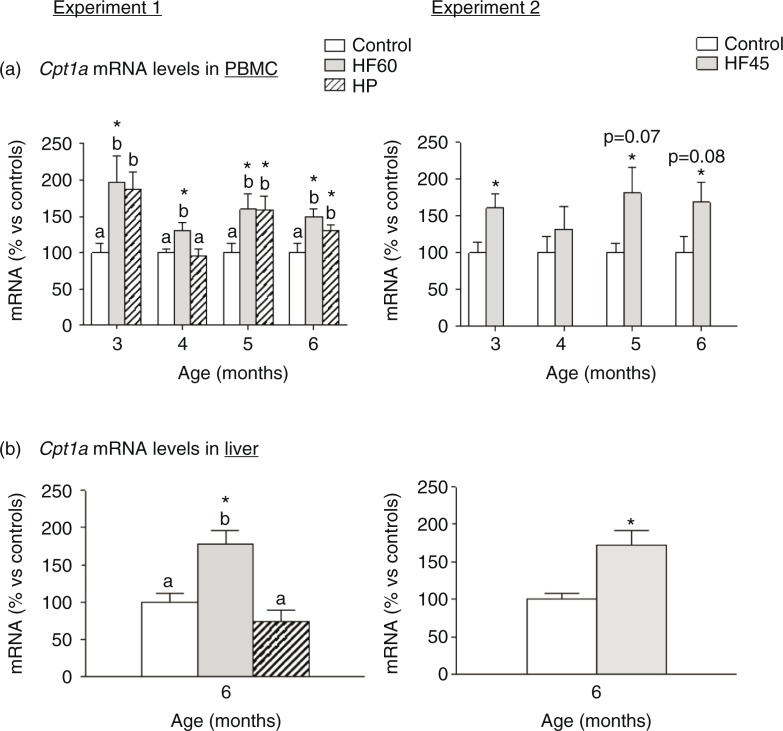
*Cpt1a* mRNA gene expression in PBMC (a) in animals of different ages and (b) in liver of 6-month-old animals. *Cpt1a* mRNA was measured by real-time RT-PCR in animals fed a control, an HF60 or an HP diet (Experiment 1), or a control or an HF45 diet (Experiment 2). Diets were administered to 2-month-old Wistar rats during 4 months. HF and HP diets were offered in isocaloric amounts to control animals and water was offered *ad libitum*. Results represent mean±SEM (*n*=5–7) of ratios of specific mRNA levels relative to *integrin beta 1* in PBMC and *beta-actin* in liver, and expressed as a percentage of the value of control group at the age of 3 (for PBMC) or 6 months (for liver) that was set to 100%. Values not sharing a common letter (a, b) are significantly different (one-way ANOVA, *p*<0.05); no letter indicates no significant differences; *indicates values significantly different versus control animals (Student's *t*-test, *p*<0.05 or indicated when different).

We analysed mRNA expression of different key genes involved in energy homeostasis in PBMC obtained monthly since the beginning of the dietary intervention, but the most outstanding results were those of the key lipolytic gene carnitine palmitoyltransferase 1a (*Cpt1a*), which codes for an enzyme that allows entrance of fatty acids into the mitochondria for their beta-oxidation. In Experiment 1, the two unbalanced diets, rich in fat (HF60) and rich in protein (HP), produced an up-regulation of *Cpt1a* mRNA levels in PBMC obtained at the different months analysed (Fig. 5a). In Experiment 2, the HF45 diet also increased PBMC *Cpt1a* mRNA expression, although statistical significance was not reached in all the months (Fig. 5a), evidencing the relevance of the lower fat proportion. This nutritional regulatory pattern observed in PBMC of rats fed with HF diets coincided to that observed in liver at the end of the experimental period: *Cpt1a* mRNA levels increased in liver of the HF60 and HF45 groups. However, contrary to what observed in PBMC, no increased *Cpt1a* expression was observed in liver of the HP group (Fig. 5b).

### Regulation of key energy homeostatic genes in PBMC of rats fed with different unbalanced diets rich in fat or protein

**Fig. 6:**
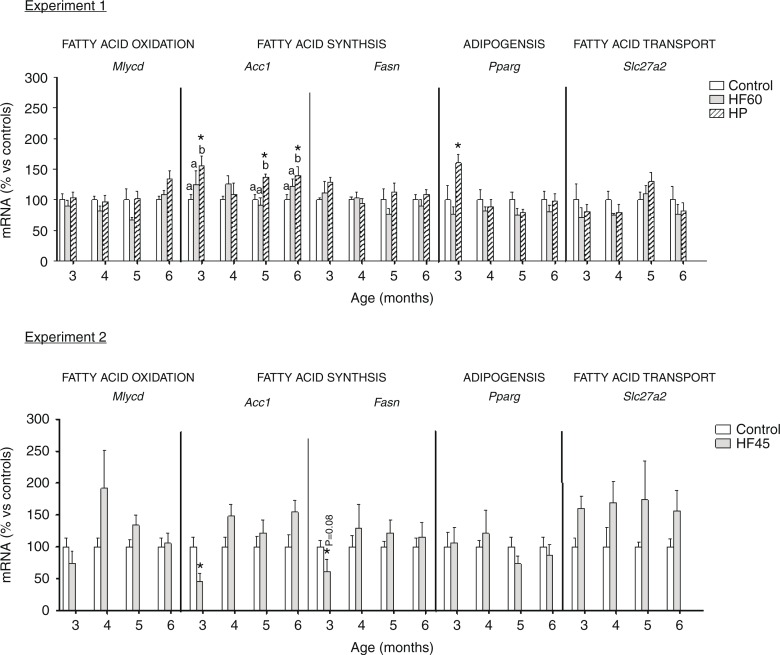
Gene expression (mRNA) of genes involved in fatty acid oxidation (*Mlycd*), fatty acid synthesis (*Acc1* and *Fasn*), adipogenesis (*Pparg*) and fatty acid transport (*Slc27a2*) in PBMC of the same animals and conditions described in Fig. 1, measured by real-time RT-PCR. Results represent mean±SEM (*n*=5–7) of ratios of specific mRNA levels relative to *integrin beta 1*, expressed as a percentage of the value of control group at the age of 3 months that was set to 100%. Values not sharing a common letter (a, b) are significantly different (one-way ANOVA, *p*<0.05); no letter indicates no significant differences; *indicates values significantly different versus control animals (Student's *t*-test, *p*<0.05 or indicated when different).

In addition to *Cpt1a*, we also analysed the effect of the unbalanced diets on PBMC expression of other relevant genes involved in lipid metabolism and, in general, no relevant effect was observed (see Fig. 6). Contrary to what happened for *Cpt1a*, dietary treatment did not affect the mRNA expression of another gene involved in fatty acid oxidation, *Mlycd*. This gene codes for malonyl-CoA decarboxylase, which catalyses the breakdown of malonyl-CoA into acetyl-CoA and carbon dioxide, inhibiting fatty acid oxidation. We also analysed the mRNA expression of two genes involved in fatty acid synthesis: *Acc1*, coding for acetyl-CoA carboxylase which catalyses the carboxylation of acetyl-CoA to produce malonyl-CoA, and *Fasn*, coding for fatty acid synthase that forms fatty acids using malonyl-CoA. Moreover, we studied mRNA expression of *Pparg* which codes for a key adipogenic factor PPAR-gamma. HP diet increased *Acc1* and *Pparg* expression in PBMC obtained at different months, the latter only after the first month of diet. However, HF diets did not affect *Acc1* or *Pparg* mRNA levels. Regarding *Fasn*, its PBMC expression was not affected by any of the unbalanced diets. No effect was observed for the expression of *Slc27a2*, coding for a fatty acid transport gene which we have reported to be affected, in PBMC, by the intake of cafeteria diets (20).

**Fig. 7:**
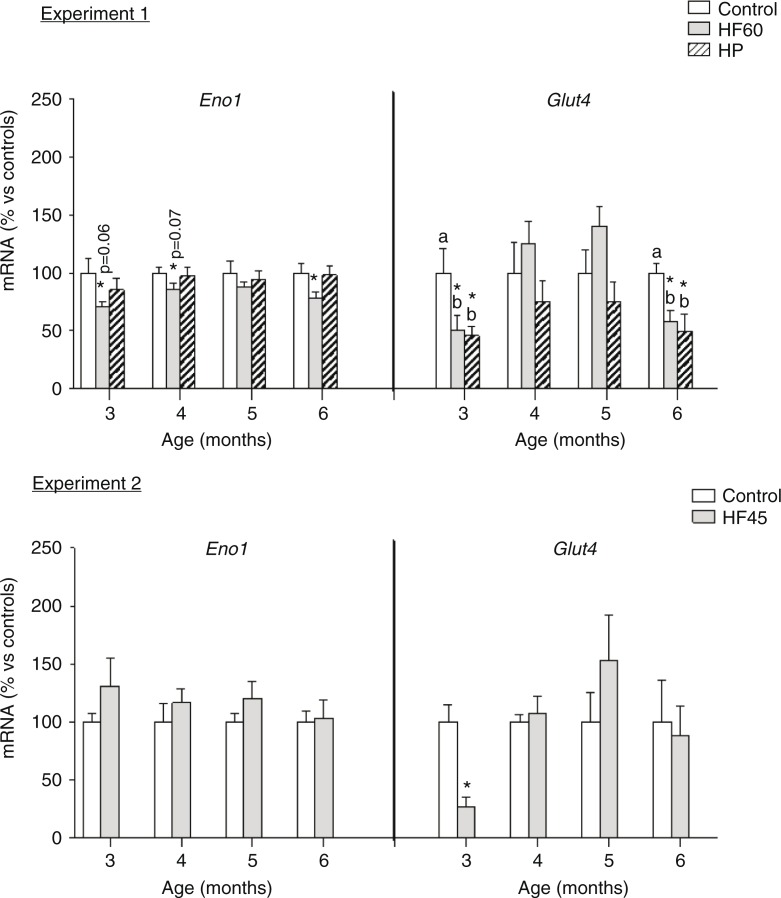
Gene expression (mRNA) of the glycolytic gene *Eno1* and of the glucose transporter *Glut4* in PBMC of the same animals and conditions described in Fig. 1, measured by real-time RT-PCR. Results represent mean±SEM (*n*=5–7) of ratios of specific mRNA levels relative to *integrin beta 1*, expressed as a percentage of the value of control group at the age of 3 months that was set to 100%. Values not sharing a common letter (a, b) are significantly different (one-way ANOVA, *p*<0.05); no letter indicates no significant differences; *indicates values significantly different versus control animals (Student's *t*-test, *p*<0.05 or indicated when different).

Finally, we analysed the potential effects of the diets in PBMC expression of two key genes involved in the maintenance of glucose homeostasis: *Eno1* and *Glut4*, coding for the glycolytic enzyme enolase 1 and for the insulin-responsive glucose transporter, respectively (see Fig. 7). *Eno1* mRNA expression resulted affected only by the HF60 diet, which decreased PBMC mRNA levels of the glycolytic gene at different months of dietary treatment; no effect was observed for the HF45 or the HP diets. *Glut4* expression in PBMC was affected by the HF60 and HP diets; both of them decreased its mRNA levels, but the effect was only observed in the first and last month of treatment, while no effect was observed for the HF45 diet. Thus, this gene was not so relevant to be considered a biomarker as the previously mentioned *Cpt1a*, whose expression was affected by all the dietary treatments and at the different time-points analysed.

### Correlation of Cpt1a mRNA in PBMC and liver with serum and liver parameters

To have a better comprehension of the relation of *Cpt1a* gene expression changes with the metabolic status of the animals, we performed Pearson correlation analysis between *Cpt1a* mRNA levels in PBMC and liver with body weight, adiposity and the measured indicators of glucose and lipid metabolism. The most relevant correlations are shown in Table 1. To be able to compare the correlations obtained for *Cpt1a* mRNA expression in PBMC with that of liver, we considered parameters obtained at the final time-point (6-month-old animals that followed 4 months of dietary treatment). Statistical correlations were found mainly taking into account HF60 and control animals from Experiment 1. *Cpt1a* mRNA expression in PBMC correlated positively with HOMA- IR index, and with TG and total lipid content of liver. Interestingly, these correlations were maintained even after considering fat mass (which was higher in the HF60 group) as a confounding factor. It is worth to note that the positive correlation between HOMA-IR index and *Cpt1a* mRNA in PBMC was also observed when using data obtained during the experiment (at 4 and 6 months of age), and also a negative correlation with TG was seen when considering all the data – from animals of 3, 4, 5 and 6 months of age – (*r*=−0.348, *p*=0.013). Surprisingly, correlations were less evident for liver *Cpt1a* mRNA expression; we observed a positive correlation with hepatic TG content and a negative correlation with circulating TG, but no relation was found with HOMA-IR or total lipid content. The effect of the HF diet on *Cpt1a* expression was dependent on the percentage of fat, as no significant correlation was found for the above mentioned parameters using data of HF45 and control animals (Experiment 2). No statistically relevant correlation was detected when considering data of the HP and control animals in Experiment 1.

**Table 1:** Correlations of *Cpt1a* mRNA expression in PBMC and liver with HOMA-IR and lipid parameters, either considering fat as a confounding factor or not

	PBMC		Liver	
		
	*Cpt1a*	*Cpt1a*	*Cpt1a*	*Cpt1a*
		
Confounding factor	None	Fat	None	Fat
Experiment 1 (data of the HF60 and control groups)				
HOMA-IR	*r*=0.671*	*r*=0.692*	*R*=0.502	*R*=0.477
	*p*=0.024	*p*=0.027	*P*=0.116	*P*=0.164
Circulating TG	*r*=−0.431	*r*=−0.325*	*R*=−0.660*	*R*=−0.601*
	*p*=0.124	*p*=0.023	*P*=0.010	*P*=0.030
Liver TG content	*r*=0.670*	*r*=0.602*	*R*=0.559*	*R*=0.488
	*p*=0.012	*p*=0.038	*P*=0.031	*P*=0.107
Liver lipid content	*r*=0.748**	*r*=0.580+	*R*=0.487	*R*=0.335
	*p*=0.005	*p*=0.061	*P*=0.126	*P*=0.315
Experiment 2 (data of the HF45 and control groups)				
HOMA-IR	*r*=−0.408	*r*=−0.062	*R*=0.153	*R*=−0.213
	*p*=0.315	*p*=0.896	*P*=0.673	*P*=0.583
Circulating TG	*r*=0.318	*r*=−0.545	*R*=−0.570	*R*=−0.566
	*p*=0.487	*p*=0.263	*P*=0.085	*P*=0.112
Liver TG content	*r*=0.386	*r*=0.735	*R*=0.425	*R*=0.381
	*p*=0.393	*p*=0.096	*P*=0.220	*P*=0.312
Liver lipid content	*r*=−0.250	*r*=0.682	*R*=0.339	*R*=−0.337
	*p*=0.550	*p*=0.092	*P*=0.308	*P*=0.341

*r*=Pearson correlation coefficients

+ *p=*0.06

* *p*<0.05

** *p*<0.01; HOMA-IR, homeostatic model assessment for insulin resistance; PBMC, peripheral blood mononuclear cells, TG, triacylglycerol.

## Discussion

The use of PBMC as a tool to identify nutritional biomarkers is increasing, since this fraction of blood cells has been validated as a good model for dietary intervention studies (17). Our group has previously reported, based on microarray analysis, that PBMC gene expression is affected by the intake of diets with an unbalanced macronutrient composition (14). Here, we aimed to identify, in PBMC, early gene expression biomarkers of diet-related metabolic alterations (mainly those linked to metabolic syndrome and liver steatosis) due to the intake of unbalanced diets (rich in fat or protein). We focused our attention on identifying early biomarkers of normal-weight obesity caused by either an increase in dietary fat intake, but without exceeding caloric needs, or by increased protein intake, simulating the relative overconsumption observed in humans following HP weight-loss diets.

We performed a first experiment (Experiment 1) in which we administered two unbalanced diets: HF (with 60% Kcal as fat vs. 10% in controls) and HP (45% Kcal as proteins vs. 20% in controls), during 4 months to adult Wistar rats. Both diets were administrated in isocaloric conditions to the control diet mainly to avoid hyperphagia and development of overweight/obesity in the HF group. Thus, animals of the HF60 group progressively developed increased adiposity (mainly visceral), due to the higher input of dietetic fat, but did not exhibit overweight. A high level of visceral fat is the principal feature of MONW subjects and is directly associated with alterations in glucose metabolism and other parameters related to metabolic syndrome (34). Accordingly, HF60 animals presented higher serum glucose levels from the first month of intake of the HF diet. However, in spite of the hyperglycaemia, circulating insulin levels were lower the first 2 months of HF diet intake, probably because of the lower carbohydrate content of the diet, as it has been previously shown that low-carbohydrate/HF diets generate hypoinsulinemia (35). Nevertheless, as a result of the sustained intake of the HF diet, animals progressively developed insulin resistance, evidenced by a higher HOMA-IR index at the final point, after 4 months of HF diet feeding. All these alterations evidence an impairment in glucose metabolism and insulin signalling due to increased fat intake, a well-known effect of HF-diet-induced obesity (36), and which is also characteristic of MONW subjects (37). Alterations were also evident for circulating TG; HF60 diet intake produced a decrease in circulating TG from the first month of diet intake until the end of the experiment. These decreased serum TG levels, which are characteristic of HF-diet interventions, have been related to a reduced very low-density lipoprotein production rate and increased TG removal from blood to be used as a source of energy because of the lower carbohydrate content of the diet (28, 38, 39). Other relevant indicators of lipid metabolism, such as circulating NEFA, did not present any significant changes in the HF group at any of the different times analysed.

The HF60 diet was particularly rich in fat; therefore, to increase the robustness of the results, we designed another experiment (Experiment 2) with a new group of rats isocalorically fed with an HF diet containing a lower amount of lipids (45% Kcal of total calories). Development of an MONW phenotype due to sustained intake of this more moderate HF diet would be more similar to the situation which could be occurring in humans. Logically, the alterations found in HF45 animals were less marked than those of the MONW HF60 group. As expected, animals presented the same body weight than controls but, in this case, 4 months of isocaloric intake of the HF45 diet was not enough to increase fat mass of the animals. However, animals presented some alterations such as increased serum glucose levels, which were observed after only 1 month of dietary intake. In spite of this, HOMA-IR index (measured after 2 and 4 months of diet intake) was not affected. As seen in the HF60 animals, serum TG levels also decreased at different time points.

Regarding the protein-rich diet, its intake resulted in a progressive decrease in body weight which was not due to a decreased fat mass, which remained unchanged during the experimental trial, but to a lower water content. This HP diet produced a lower alteration in the analysed indicators of glucose and lipid metabolism than the fat-rich diets. We did not observe alterations in serum glucose levels, NEFA or TG and, although higher insulin levels were required to maintain glycaemia in HP-fed animals after 4 months of dietary treatment, HOMA-IR remained unchanged.

One of the most, somehow unexpected, relevant features observed in our three animal models was an alteration in liver lipid composition. Liver is a key tissue, which is involved in energy balance maintenance by appropriate handling of ingested macronutrients (40), and increased TG deposition in this tissue has been associated with a higher risk of metabolic syndrome, different hepatic pathologies and death (27, 41). Notably, we found an increased TG content in liver of the different dietary groups studied: HF60, HF45 and HP. We have previously described in detail, based on microarray data analysis, the potential mechanisms involved in TG accumulation in liver due to the intake of isocaloric HF (42) or HP diets (article submitted). Because fatty liver is a serious health concern, it would be of great interest to identify easily obtainable non-invasive biomarkers of increased fat accumulation in this relevant organ, which could help to predict future metabolic diseases.

To deal with the identification of PBMC biomarkers, and based on our previous experience showing that PBMC reflect with particular accuracy lipid metabolism gene expression in response to nutritional interventions (19, 20, 22, 23), we selected key genes involved in fatty acid oxidation (*Cpt1a* and *Mlycd*), fatty acid synthesis (*Acc1* and *Fasn*), adipogenesis (*Pparg*) and fatty acid transport (*Slc27a2*), to be tested as potential early markers of health risk related to dietary interventions. In addition, the HF and HP diets, as those used in our experiment, usually imply a decrease in carbohydrate content; therefore, we also analysed the expression of a key glycolytic gene (*Eno1*) and of a relevant glucose transporter (*Glut4*) to be considered as potential early biomarkers of dietary imbalance in PBMC. We analysed these genes in PBMC samples obtained monthly from the different animal models during the 4 months of the dietary intervention.

The most outstanding results were those obtained for *Cpt1a*, involved in long-chain fatty acids transport across the inner mitochondrial membrane. We had previously reported that *Cpt1a* mRNA expression in PBMC is a good indicator of gene expression patterns typical of key energy homeostatic tissues such as liver and adipose tissues in response to changes in dietary patterns (fasting/refeeding) or *ad libitum* intake of HF diets (19, 22, 24). Interestingly, our data show that *Cpt1a* mRNA levels increased in animals isocalorically fed with the two different HF diets (HF60 and HF45) and also in animals fed with the HP diet. The increase was observed after only 1 month of the dietary treatment, when animals had not yet developed changes in body weight, fat mass or HOMA-IR index. This increase in *Cpt1a* expression was also observed in the following 3 months of the experimental trial. In the case of the HF groups, increased *Cpt1a* expression, which was statistically stronger in the HF60 than in the HF45 group, reflected higher levels of this gene in liver, and thus evidenced the expected higher fatty acid oxidative capacity. However, the sustained increase in *Cpt1a* expression observed in PBMC of the HP group did not correlate with an increased expression in liver of the animals.

To have a better comprehension of the utility of *Cpt1a* expression analysis in PBMC as a dietary biomarker of health risk, we performed correlations with the different analysed parameters at the end of the experiment. Relevant correlations were obtained considering the HF60 animals (those that presented strongest metabolic alterations) and controls. Interestingly, we found positive correlations between *Cp1a* mRNA in PBMC and HOMA-IR, liver TG and total liver lipid content, as well as a negative correlation with circulating TG. These correlations were maintained even after correcting by fat mass (which was higher in HF60 animals than in controls) as a confounding factor. This is relevant, since altered (increased) *Cpt1a* expression in PBMC of MONW animals is not only a reflection/consequence of higher adiposity in the animals but also provides additional information, such as increased risk of developing insulin resistance or accumulating fat in liver. Moreover, analysis of *Cpt1a* analysed in PBMC provided better correlations than in liver; hepatic *Cpt1a* mRNA expression, taking into account HF60 and control animals, correlated positively with liver TG content, but not with total liver lipid content or HOMA-IR. No relevant correlations were obtained in PBMC or liver in the case of HF45 or HP animals, probably due to the fact that the alterations observed in these animals were not as strong as those in HF60 ones. However, all the groups presented higher TG accumulation in liver (i.e. increased metabolic risk) at the end of the experiment as well as increased PBMC *Cpt1a* expression not only at the final point, but since the first month of unbalanced dietary treatment. Thus, based on the observed correlations, we could consider this gene as an early risk marker of diet-related liver damage and insulin resistance, of interest to identify dietary imbalances that can affect health, particularly lipid accumulation in liver, when animals are still healthy.

Gene expression analysis of the rest of pre-selected potential biomarkers did not report relevant results. It is well known that PBMC can reflect metabolic adaptations to the intake of HF/high energy diets by inhibition of lipogenic and enhancement of lipolytic genes (19, 22, 24). Interestingly, in our study, apart from *Cpt1a*, the other lipid metabolism-related genes were not importantly affected, probably because our diets were offered in isocaloric conditions to controls, not *ad libitum*. This is an additional evidence of the robustness of *Cpt1a* as a biomarker, as its gene expression reflected expected liver behaviour in response to a lighter dietary stimulus: isocaloric HF diets non-related to overweight. In addition, we observed changes in gene expression of the two studied genes involved in glucose homeostasis, the glycolytic gene *Eno1* and the insulin-responsive glucose transporter *Glut4*. According to the lower carbohydrate content of the diet, *Eno1* mRNA levels decreased at different ages, but the decrease did not always reach statistical significance and was only observed in the HF60 group. On the other hand, *Glut4* expression greatly decreased in the HF and HP groups, but only the first and the last month of dietary treatment, while *Cpt1a* altered expression was observed at all the different months studied.

In summary, in HF-diet pair-fed animals that developed an MONW phenotype, *Cpt1a* gene expression increased in PBMC, reflecting the nutritional response that takes place in liver aimed to increase the usage of fatty acids as energy and preserve glucose, as an adaptation to the higher fat and decreased carbohydrate content of the diet. These results evidence the usefulness of PBMC as a surrogate tissue to study nutritional effects on other internal organs. Moreover, the same adaptation (increased *Cpt1a* expression) was observed in PBMC of HP-fed animals, even if *Cpt1a* did not increase in liver of these animals. These higher *Cpt1a* mRNA levels were observed after only 1 month of dietary intervention with both unbalanced diets and were maintained thereafter. Thus, *Cpt1a* gene expression analysis in PBMC could be used as a blood marker of dietary macronutrient intake to report nutritional imbalances. The use of molecular dietary biomarkers provides a more objective and accurate measure of intake in comparison to traditional questionnaires used in dietetic studies and will improve the assessment of the relationship between diet and chronic metabolic alterations or diseases. *Cpt1a* expression in PBMC positively correlated with HOMA-IR and with liver fat and TG content using data of HF60 MONW animals (those presenting the strongest metabolic alterations). Based on this, the results of this work suggest that *Cpt1a* can be used, not only as a nutritional biomarker, but as an early marker of future alterations such as liver fat deposition, which is a common feature of animals fed with both the HF and the HP unbalanced diets. At present, development of overweight and alterations of classical blood parameters are used to identify metabolic syndrome. However, a high percentage of the population currently present a high risk to develop metabolic syndrome in the future, even with a normal BMI (11, 43). Moreover, according to our data, unbalanced diets can result in increased metabolic syndrome risk (measured as fat liver accumulation), not only in the absense of overweight/obesity but also in the absense of increased adiposity or signs of insulin resistance, as seen in the animals fed with the moderate HF diet (HF45) or the HP diet. For this reason, it is important to identify robust early markers which could help detect dietary imbalances and therefore act in consequence.

## Conclusions

Our results reinforce the use of PBMC as a tool in nutritional studies and clearly show that *Cpt1a* analysis in PBMC could be used as an early biomarker of future complications related to the intake of unbalanced diets, such as insulin resistance and increased liver fat deposition. At this point, it would be of interest to confirm the usefulness of this biomarker in humans.

## Supplementary Material

*Cpt1a* gene expression in peripheral blood mononuclear cells as an early biomarker of diet-related metabolic alterations
