# Supplementary material for: Cpt1a gene expression in peripheral blood mononuclear cells as an early biomarker of diet-related metabolic alterations
Source: Food Nutr Res. 2016 Nov 23;60:10.3402/fnr.v60.33554. doi: 10.3402/fnr.v60.33554 (PMC5123217; doi:10.3402/fnr.v60.33554)
Supplement: Cpt1a gene expression in peripheral blood mononuclear cells as an early biomarker of diet-related metabolic alterations [file FNR-60-33554-s001.docx]

| Gene | Forward primer  (5’ to 3’) | Reverse primer  (5’ to 3’) | Product  size (bp) |
| --- | --- | --- | --- |
| **Fatty acid oxidation** |  |  |  |
| *Cpt1a* | GCAAACTGGACCGAGAAGAG | CCTTGAAGAAGCGACCTTTG | 180 |
| *Mlycd* | GGGGCTGTGATGTGGCGTAT | GGTAGGAGATGCTGTTGGGGC | 127 |
| **Fatty acid synthesis** |  |  |  |
| *Acc1* | TGCAGGTATCCCCACTCTTC | TTCTGATTCCCTTCCCTCCT | 212 |
| *Fasn1* | CGGCGAGTCTATGCCACTAT | ACACAGGGACCGAGTAAT | 222 |
| **Adipogenesis** |  |  |  |
| *Pparg* | AGAGCCTTCAAACTCCCTCA | GAGACATCCCCACAGCAAG | 230 |
| **Fatty acid transport** |  |  |  |
| *Slc27a2* | CTGAAAAAGGAGGGCGTGT | TATGTAGACTGCGGGTGTGG | 156 |
| **Glucose homeostasis** |  |  |  |
| *Eno1* | ACCCTCTTTCCTTCCTCCGCA | CGCAGCACGGAAGAGACCTTT | 148 |
| *Glut4* | CAGGGCAAGGATGGTAGAGCAC | TCCCCTCCTGCCTTAGTTGGTC | 149 |
| **Reference genes** |  |  |  |
| *Actb* | TACAGCTTCACCACCACAGC | TCTCCAGGGAGGAAGAGGAT | 120 |
| *Itgb1* | TTGTGGGTCGCTGATTGGCT | CTCCAGCAAAGTGAAACCCAGC | 80 |

**Supplementary Table 1.** Nucleotide sequences of primers and amplicon size used for real-time RT-PCR amplification.

For genes related to fatty acid oxidation: *Cpt1a*, carnitine palmitoyltransferase 1a; and *Mlycd,* malonyl-CoA decarboxylase. For genes related to fatty acid synthesis: *Acc1*, acetyl-CoA carboxylase 1; *Fasn*, fatty acid synthase; and *Pparg*, peroxisome proliferator-activated receptor gamma. For fatty acid transport: *Slc27a2*, solute carrier family 27 (fatty acid transporter), member 2. For genes related to carbohydrate metabolism: *Eno1*, enolase 1; and *Glut4*, glucose transporter 4. Reference genes: *Actb*, beta-actin; and *Itgb1*, integrin, beta 1.
